# Machine learning models integrating intracranial artery calcification to predict outcomes of mechanical thrombectomy

**DOI:** 10.3389/fneur.2025.1642807

**Published:** 2025-08-06

**Authors:** Guangzong Li, Yuesen Zhang, Di Li, Manhong Zhao, Lin Yin

**Affiliations:** ^1^Department of Neurology, The Second Affiliated Hospital of Dalian Medical University, Dalian, China; ^2^Department of Neurointervention and Neurocritical Care, The Central Hospital Affiliated to Dalian University of Technology, Dalian, China

**Keywords:** intracranial artery calcification, mechanical thrombectomy, ischemic stroke, artificial intelligence, machine learning

## Abstract

**Objective:**

To investigate whether intracranial artery calcification (IAC) serves as a reliable imaging predictor of mechanical thrombectomy (MT) outcomes and to develop robust machine learning (ML) models incorporating preoperative emergency data to predict outcomes in patients with acute ischemic stroke (AIS).

**Methods:**

This retrospective study included patients with AIS and anterior circulation occlusion who underwent MT at the Second Affiliated Hospital of Dalian Medical University and the Central Hospital Affiliated to Dalian University of Technology between January 2017 and December 2024. Patients were categorized into favorable [modified Rankin Scale (mRS) 0–2] and poor outcome (mRS 3–6) groups based on their 90-day functional independence. Preoperative clinical and radiological data, including a quantitative assessment of IAC, were systematically collected. Eleven ML algorithms were trained and validated using Python, and external validation and performance evaluations were conducted. The Shapley additive explanation (SHAP) method was used to interpret the optimal model.

**Results:**

A total of 823 eligible patients were enrolled and stratified into training (*n* = 437), internal validation (*n* = 188), and external testing (*n* = 198) cohorts. The Extra Trees model demonstrated the highest predictive accuracy. The top three predictors were a history of hypertension, serum albumin level, and total calcified volume.

**Conclusion:**

The total volume of IAC is a critical imaging biomarker for predicting MT outcomes in patients with anterior circulation AIS. The ML models developed using preoperative emergency data demonstrated strong predictive performance, providing a valuable tool to help clinicians identify suitable MT candidates with greater precision.

## 1 Introduction

With advancements in neurointerventional techniques–particularly after the publication of five large randomized controlled trials in the *New England Journal of Medicine* in 2015–mechanical thrombectomy (MT) has become the standard treatment for acute-phase anterior circulation acute ischemic stroke (AIS) (1–5). However, meta-analyses show that only 46% of patients achieve favorable outcomes, with a significant proportion of patients still experiencing poor outcomes or death (6). This underscores the urgent need for reliable predictive models to identify appropriate candidates for MT. Several traditional scoring systems have been developed using logistic regression to predict MT outcomes. These include: The Pittsburgh Response to Endovascular therapy (PRE) score (7), which considers age, National Institutes of Health Stroke Scale (NIHSS), and Alberta Stroke Program Early CT Score (ASPECTS); The Stroke Prognostication using Age and NIH Stroke Scale (SPAN) score (8), based on age and NIHSS; The Totaled Health Risks in Vascular Events (THRIVE) score (9), which includes age, NIHSS, and chronic comorbidities; and The Houston Intra-Arterial Therapy (HIAT) score (10), which incorporates age, NIHSS, and admission blood glucose, and its updated version, HIAT2 (11), which also includes ASPECTS. The area under the receiver operating characteristic (ROC) curve for these scores ranges from 0.56 to 0.79, highlighting the need for improved predictive accuracy.

Machine learning (ML) enables high-precision analysis of large clinical datasets using artificial intelligence algorithms. A recent meta-analysis of MT outcome prediction models found that machine learning-based models generally outperform traditional models in predicting the outcomes of neurointerventional procedures, while emphasizing the importance of external validation for model generalizability. However, existing machine learning models for predicting MT outcomes are predominantly based on post-procedural clinical data (12–14). Only a limited number of studies have incorporated only preprocedural clinical data and the simple imaging score ASPECTS, generally overlooking the key imaging features of arterial calcified plaques (15). Intracranial artery calcification (IAC), a common imaging finding on non-contrast cranial computed tomography (CT), is closely associated with atherosclerotic plaques and potentially exerts a more direct impact on MT outcomes (16). Although previous studies have explored the association between IAC and MT outcomes, their conclusions have shown significant discrepancies (17, 18). Therefore, we conducted a comprehensive quantitative assessment of IAC and evaluated its potential as an imaging biomarker for MT prognosis. Additionally, we aimed to develop machine learning models using emergency preprocedural data to aid in the precise identification of AIS patients most likely to benefit from MT.

## 2 Methods

### 2.1 Data source

We retrospectively enrolled patients with anterior circulation AIS who underwent MT at the Second Affiliated Hospital of Dalian Medical University and the Central Hospital Affiliated to Dalian University of Technology between January 2017 and December 2024. This study received ethical approval from the Institutional Ethics Committee (approval number: KY2025-014-01), with a waiver of informed consent granted due to the retrospective nature of the study.

#### 2.1.1 Inclusion and exclusion criteria

Inclusion criteria: (1) Age ≥18 years; (2) Availability of baseline non-contrast cranial computed tomography (CT) scans; (3) Signed informed consent for MT treatment of anterior circulation occlusion. Exclusion criteria: (1) severe cardiopulmonary or renal dysfunction, (2) incomplete follow-up data, (3) poor-quality cranial CT images, and (4) pre-stroke modified Rankin Scale (mRS) score >2.

### 2.2 Data collection

Demographic information, preoperative emergency clinical parameters, and baseline non-contrast CT scans were systematically collected. Clinical variables included medical history of hypertension, diabetes mellitus, coronary artery disease, atrial fibrillation, previous stroke history, smoking, alcohol consumption, tumor, intravenous thrombolysis, advanced imaging, laterality of the occluded vessel, NIHSS score, activated partial thromboplastin time, prothrombin time, international normalized ratio, fibrinogen, thrombin time, white blood cell count, neutrophil count, lymphocyte count, neutrophil-to-lymphocyte ratio, red blood cell count, hemoglobin, platelet, urea, creatinine, blood glucose, and albumin. Functional outcomes were assessed via telephone follow-up at 90 days post-stroke using the mRS, evaluated by a neurologist blinded to clinical data. Patients were classified into favorable (mRS 0–2) and poor (mRS 3–6) outcome groups.

### 2.3 IAC quantification

**Figure 1:**
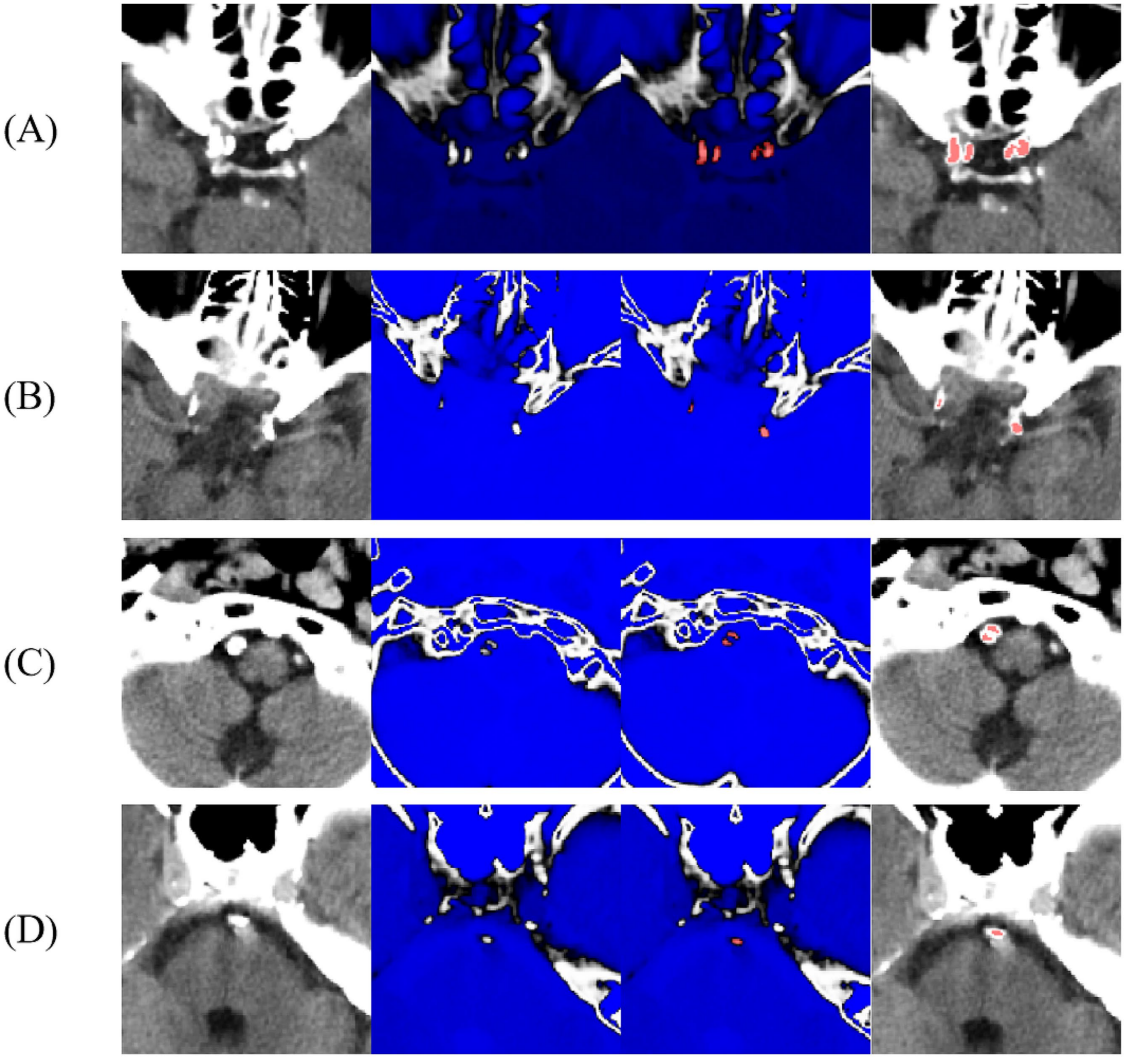
Schematic diagram of intracranial artery calcification segmentation **(A–D)** internal carotid artery, middle cerebral artery, vertebral artery, and basilar artery; blue indicates CT values below the 130 HU threshold defined by ITK-SNAP software.

All pre-MT CT scans were acquired using Siemens 64-slice CT scanners (120 kV, 260 mAs, 5 mm slice thickness, and coverage from the external auditory meatus to the cranial vertex). Calcifications were defined as hyperdense lesions ≥130 Hounsfield Units (HU) spanning ≥2 contiguous voxels. IAC quantification referred to the previously described and validated methods (19, 20). Two experienced neurologists with over 10 years of expertise in neurointervention independently used ITK-SNAP software to set a CT value threshold of 130 Hounsfield Units (HU) to delineate regions of interest (ROIs)–specifically, IAC lesions, layer-by-layer on non-contrast brain CT images, without access to any patient data (Figure 1). If the volume difference between the two evaluators’ ROI exceeded 10 mm^3^, the evaluators reviewed the ROIs collaboratively, discussed discrepancies, and independently re-delineated the ROIs until the difference was within 10 mm^3^, ensuring accuracy. The final ROIs were confirmed by a senior chief physician with over 20 years of experience in neurointervention and quantitatively assessed using ITK-SNAP software. Prior to image evaluation, standardized training was conducted by two neurologists with more than 10 years of neurointerventional experience. Fifty cranial CT images were independently delineated, and inter-rater consistency was evaluated using the intraclass correlation coefficient (ICC). An ICC > 0.75 indicated excellent consistency. At the end of training, the ICC between the two evaluators was 0.92 (Supplementary Table 1). The delineated vessels included the internal carotid, middle cerebral, vertebral, and basilar arteries. Measurement indicators included the presence of calcification, total calcified volume (TCV), ipsilateral culprit-vessel calcified volume (ICV), calcified vessel count, and calcification pattern. Due to the highly skewed distribution of volumes and the inclusion of patients with zero calcification, a logarithmic transformation was applied to TCV and ICV, expressed as ln(volume + 1), with units in cubic millimeters. The classification system proposed by Kockelkoren et al. was adopted to evaluate internal carotid artery calcification patterns (21).

### 2.4 Machine learning model development and validation

Variables with <10% missing data were included in the analysis. Given this low proportion, missing values were imputed using the mean or median of the training set, depending on the data type. The Central Hospital cohort (*n* = 625) was randomly divided into training (70%, *n* = 437) and internal validation (30%, *n* = 188) sets. The cohort from the Second Affiliated Hospital (*n* = 198) served as the external test set. Feature selection was performed using least absolute shrinkage and selection operator (LASSO) regression. 5-fold cross-validation was conducted on the training set, and a random search method was employed for hyperparameter tuning. Eleven machine learning algorithms were trained using Python 3.8 and Scikit-learn: Support Vector Machine (SVM), Random Forest, Extra Trees, Extreme Gradient Boosting (XGBoost), LightGBM, Naive Bayes, Adaptive Boosting (AdaBoost), Gradient Boosting, Logistic Regression (LR), Multilayer Perceptron (MLP), and Decision Tree. Internal and external validations were performed, and model performance was evaluated using the area under the ROC curve (AUC) and decision curve analysis (DCA). The optimal model was selected through a holistic assessment of multiple performance metrics, including AUC, accuracy, sensitivity, and specificity, with statistical comparisons conducted via DeLong’s test. Model interpretability was further enhanced using Shapley Additive Explanations (SHAP).

### 2.5 Statistical analysis

All statistical analyses were conducted using Python 3.8. Normality was assessed using the Kolmogorov–Smirnov test. Continuous variables are presented as mean±SD (for normally distributed data) or median [interquartile range] (for non-normally distributed data). Categorical variables are expressed as frequencies (percentages).

## 3 Results

### 3.1 Baseline characteristics

A total of 823 patients with anterior circulation MT who met inclusion and exclusion criteria were enrolled. Of these, 276 (33.54%) were classified into the favorable outcome group and 547 (66.46%) into the poor outcome group. The cohort was stratified into training (*n* = 437), internal validation (*n* = 188), and external testing (*n* = 198) sets. Thirty-seven variables were included in the analysis; baseline characteristics are summarized in Table 1.

**Table 1:** Baseline characteristics of patients in the training set, internal validation set, and external test set.

Variables	Total (*n* = 823)	Train set (*n* = 437)	Validation set (*n* = 188)	Test set (*n* = 198)
Age, M (Q₁, Q₃)	71.00 (64.00, 78.00)	71.00 (64.00, 77.00)	70.00 (64.00, 78.00)	72.00 (65.00, 79.00)
Men, *n* (%)	527 (64.03)	278 (63.62)	129 (68.62)	120 (60.61)
Hypertension, *n* (%)	514 (62.45)	264 (60.41)	114 (60.64)	136 (68.69)
Diabetes, *n* (%)	206 (25.03)	107 (24.49)	45 (23.94)	54 (27.27)
Atrial fibrillation, *n* (%)	386 (46.90)	197 (45.08)	87 (46.28)	102 (51.52)
Coronary heart disease, *n* (%)	139 (16.89)	76 (17.39)	24 (12.77)	39 (19.70)
Previous stroke history, *n* (%)	115 (13.97)	53 (12.13)	25 (13.30)	37 (18.69)
Smoking, *n* (%)	317 (38.52)	165 (37.76)	74 (39.36)	78 (39.39)
Alcohol, *n* (%)	216 (26.25)	104 (23.80)	44 (23.40)	68 (34.34)
Tumor, *n* (%)	65 (7.90)	27 (6.18)	11 (5.85)	27 (13.64)
Intravenous thrombolysis, *n* (%)	354 (43.01)	180 (41.19)	88 (46.81)	86 (43.43)
Advanced imaging, *n* (%)	354 (43.01)	226 (51.72)	94 (50.00)	34 (17.17)
Left occlusion, *n* (%)	398 (48.36)	210 (48.05)	88 (46.81)	100 (50.51)
NIHSS, M (Q₁, Q₃)	16.00 (13.00, 20.00)	16.00 (13.00, 21.00)	17.00 (14.00, 22.00)	15.00 (13.00, 19.00)
APTT, M (Q₁, Q₃)	33.10 (30.60, 36.15)	33.10 (30.30, 36.50)	33.10 (30.48, 36.45)	33.05 (30.90, 35.48)
Prothrombin time, M (Q₁, Q₃)	13.30 (12.70, 14.00)	13.30 (12.60, 14.00)	13.30 (12.60, 14.20)	13.30 (12.80, 13.80)
International normalized ratio, M (Q₁, Q₃)	1.03 (0.99, 1.10)	1.03 (1.00, 1.12)	1.03 (1.01, 1.12)	1.02 (0.96, 1.06)
Fibrinogen, M (Q₁, Q₃)	3.20 (2.74, 3.73)	3.20 (2.71, 3.63)	3.20 (2.73, 3.64)	3.33 (2.84, 4.04)
Thrombin time, M (Q₁, Q₃)	17.60 (16.90, 18.60)	17.60 (16.90, 18.80)	17.60 (16.98, 18.83)	17.30 (16.70, 17.90)
White blood cell, M (Q₁, Q₃)	7.75 (6.27, 9.47)	7.75 (6.36, 9.51)	7.75 (6.60, 9.52)	7.44 (5.96, 9.13)
Neutrophil, M (Q₁, Q₃)	5.15 (3.85, 6.88)	5.15 (3.93, 6.82)	5.18 (4.00, 7.17)	4.79 (3.54, 6.57)
Lymphocyte, M (Q₁, Q₃)	1.63 (1.15, 2.20)	1.63 (1.15, 2.20)	1.63 (1.05, 2.13)	1.67 (1.19, 2.31)
Neutrophil lymphocyte ratio, M (Q₁, Q₃)	3.04 (2.01, 5.09)	3.04 (2.05, 5.07)	3.11 (2.21, 5.53)	2.65 (1.73, 4.80)
Red blood cell, M (Q₁, Q₃)	4.58 (4.24, 4.91)	4.58 (4.30, 4.92)	4.58 (4.26, 4.88)	4.56 (4.08, 4.88)
Hemoglobin, M (Q₁, Q₃)	142.00 (130.00, 153.00)	142.00 (133.00, 154.00)	141.50 (132.00, 151.00)	142.00 (126.25, 151.00)
Platelet, M (Q₁, Q₃)	189.00 (161.00, 221.50)	189.00 (163.00, 221.00)	189.00 (165.00, 224.75)	184.50 (153.25, 218.75)
Urea, M (Q₁, Q₃)	6.40 (5.40, 7.67)	6.40 (5.49, 7.70)	6.40 (5.59, 7.82)	6.40 (5.20, 7.35)
Creatinine, M (Q₁, Q₃)	68.00 (57.40, 81.05)	68.00 (57.00, 80.00)	68.00 (57.75, 81.00)	69.20 (59.37, 85.30)
Admission blood glucose, M (Q₁, Q₃)	7.40 (6.60, 8.91)	7.40 (6.49, 9.30)	7.40 (6.67, 9.29)	7.40 (6.77, 8.32)
Albumin, M (Q₁, Q₃)	41.60 (39.40, 43.75)	41.60 (40.00, 44.00)	41.60 (40.35, 44.10)	40.35 (38.32, 42.98)
Onset to door time, M (Q₁, Q₃)	135.00 (60.00, 240.00)	165.00 (60.00, 275.00)	165.00 (90.00, 270.00)	89.50 (53.50, 191.50)
ASPECTS, M (Q₁, Q₃)	9.00 (7.00, 10.00)	8.00 (7.00, 9.00)	8.00 (7.00, 10.00)	9.00 (8.00, 10.00)
Calcified vessel count, M (Q₁, Q₃)	2.00 (1.00, 3.00)	2.00 (1.00, 3.00)	2.00 (1.00, 3.00)	2.00 (1.00, 3.00)
In(ICV + 1), M (Q₁, Q₃)	3.74 (0.00, 4.81)	3.81 (1.50, 4.91)	3.87 (1.75, 4.84)	3.39 (0.00, 4.47)
In(TCV + 1), M (Q₁, Q₃)	4.62 (3.38, 5.54)	4.72 (3.45, 5.62)	4.84 (3.42, 5.68)	4.33 (3.25, 5.16)
Calcification, *n* (%)	706 (85.78)	375 (85.81)	166 (88.30)	165 (83.33)
90-day mRS score 0–2, *n* (%)	276 (33.54)	160 (36.61)	61 (32.45)	55 (27.78)
Internal carotid artery calcification patterns				
No calcification, *n* (%)	140 (17.01)	71 (16.25)	27 (14.36)	42 (21.21)
Intimal calcification, *n* (%)	594 (72.17)	321 (73.46)	136 (72.34)	137 (69.19)
Medial calcification, *n* (%)	29 (3.52)	18 (4.12)	6 (3.19)	5 (2.53)
Mixed calcification, *n* (%)	60 (7.29)	27 (6.18)	19 (10.11)	14 (7.07)

NIHSS, National Institutes of Health Stroke Scale; APTT, activated partial thromboplastin time; ASPECTS, Alberta Stroke Program Early CT Score; ICV, ipsilateral culprit-vessel calcified volume; TCV, total calcified volume; mRS, modified Rankin Scale.

### 3.2 Machine learning model performance

**Figure 2:**
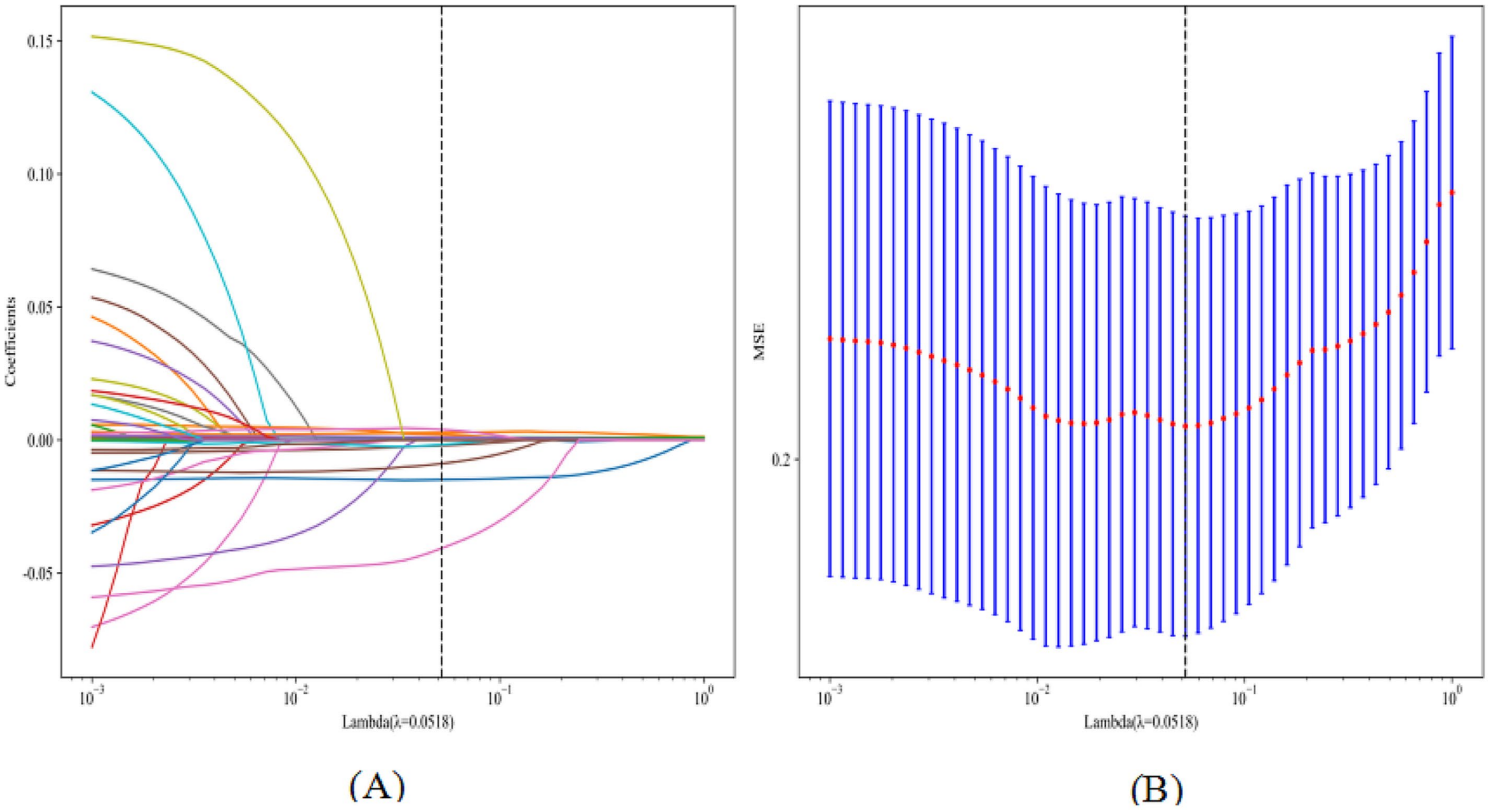
LASSO plot for feature selection (**A**, coefficient path plot; **B**, cross-validation error plot).

**Figure 3:**
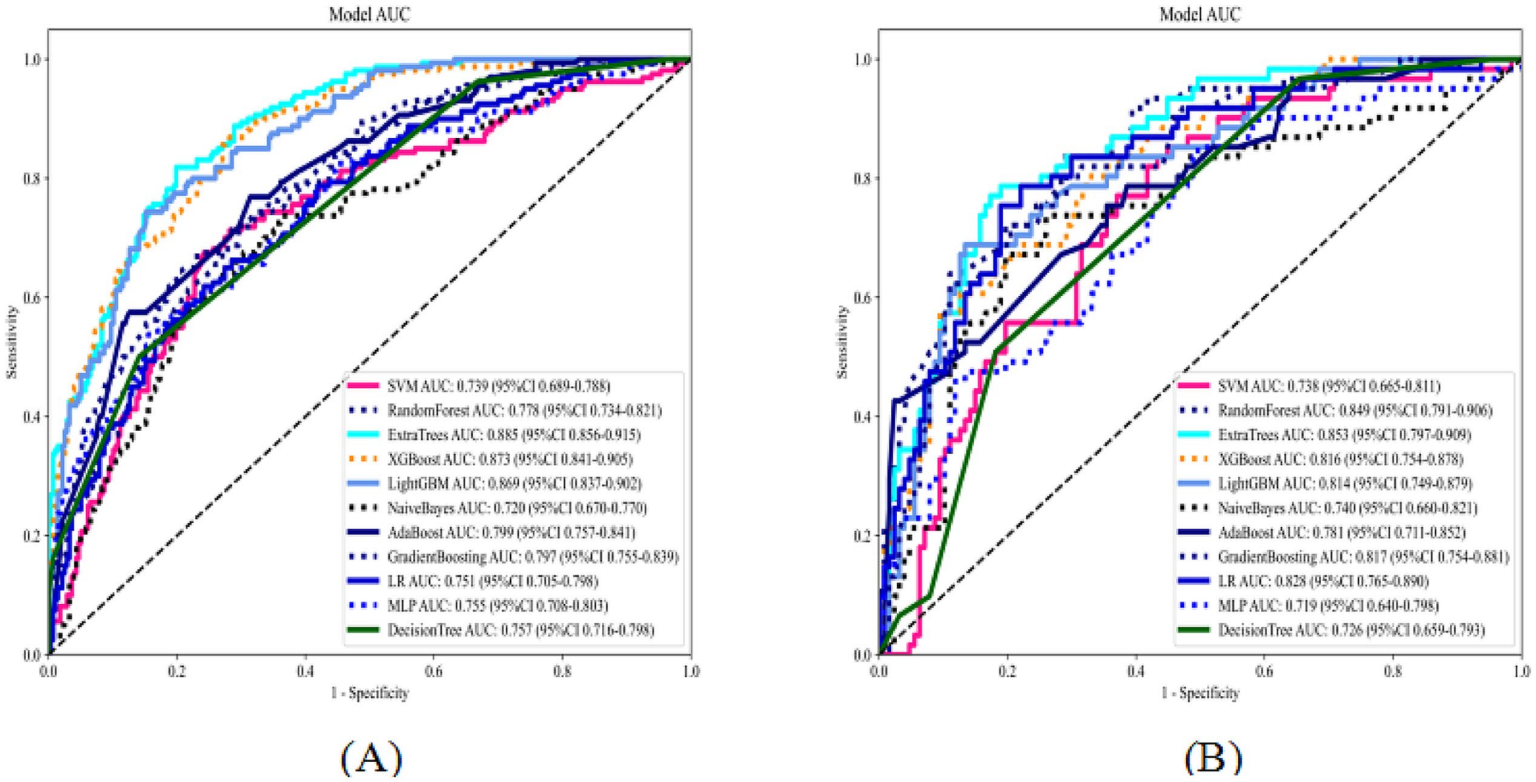
ROC curves of 11 machine learning models on the training and internal validation sets (**A**: training set; **B**: internal validation set).

**Figure 4:**
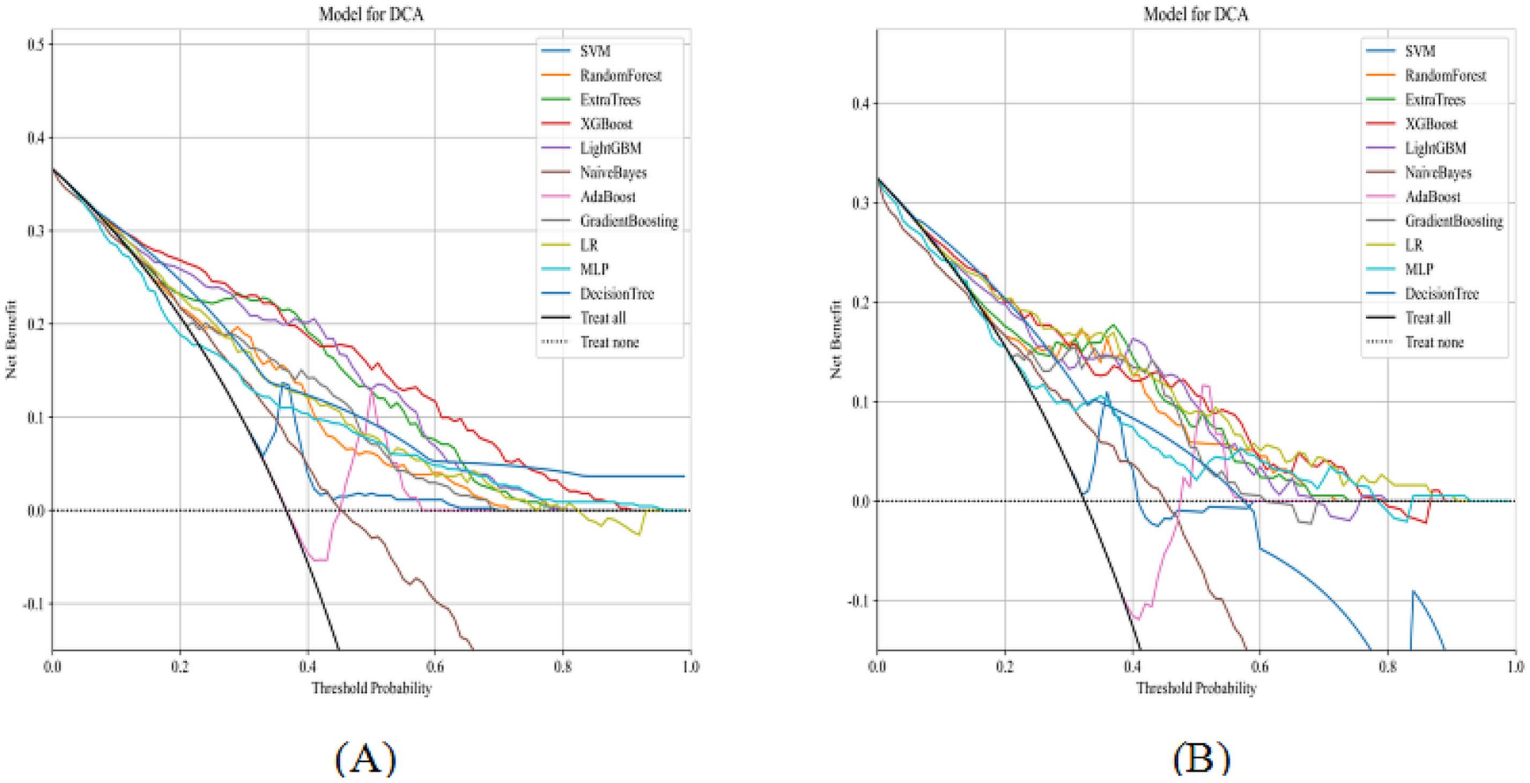
DCA of 11 machine learning models on the training and internal validation sets (**A**, training set; **B**, internal validation set).

LASSO regression with 10-fold cross-validation for regularization parameter tuning selected ten features for model development (Figure 2). Figure 3 presents ROC curves for the training and internal validation sets across the 11 machine learning models. Table 2 summarizes the AUC, accuracy, sensitivity, and specificity of each model in both sets. DCA indicated a substantial net clinical benefit across a broad threshold probability range, demonstrating strong clinical utility (Figure 4).

**Table 2:** Predictive performance of 11 machine learning models on the training and internal validation sets.

Model	Task	AUC	Accuracy	Sensitivity	Specificity
SVM	Train	0.74	0.73	0.67	0.76
SVM	Validation	0.74	0.65	0.85	0.55
RandomForest	Train	0.78	0.71	0.72	0.71
RandomForest	Validation	0.85	0.76	0.84	0.72
ExtraTrees	Train	0.89	0.81	0.82	0.80
ExtraTrees	Validation	0.85	0.80	0.79	0.81
XGBoost	Train	0.87	0.78	0.86	0.73
XGBoost	Validation	0.82	0.79	0.61	0.87
LightGBM	Train	0.87	0.81	0.74	0.85
LightGBM	Validation	0.81	0.81	0.69	0.87
NaiveBayes	Train	0.72	0.68	0.74	0.64
NaiveBayes	Validation	0.74	0.74	0.74	0.74
AdaBoost	Train	0.80	0.72	0.77	0.69
AdaBoost	Validation	0.78	0.80	0.43	0.98
GradientBoosting	Train	0.80	0.74	0.64	0.80
GradientBoosting	Validation	0.82	0.77	0.72	0.80
LR	Train	0.75	0.70	0.66	0.71
LR	Validation	0.83	0.78	0.79	0.78
MLP	Train	0.76	0.73	0.54	0.84
MLP	Validation	0.72	0.62	0.85	0.50
DecisionTree	Train	0.76	0.73	0.50	0.86
DecisionTree	Validation	0.73	0.72	0.51	0.82

### 3.3 Model interpretation

**Figure 5:**
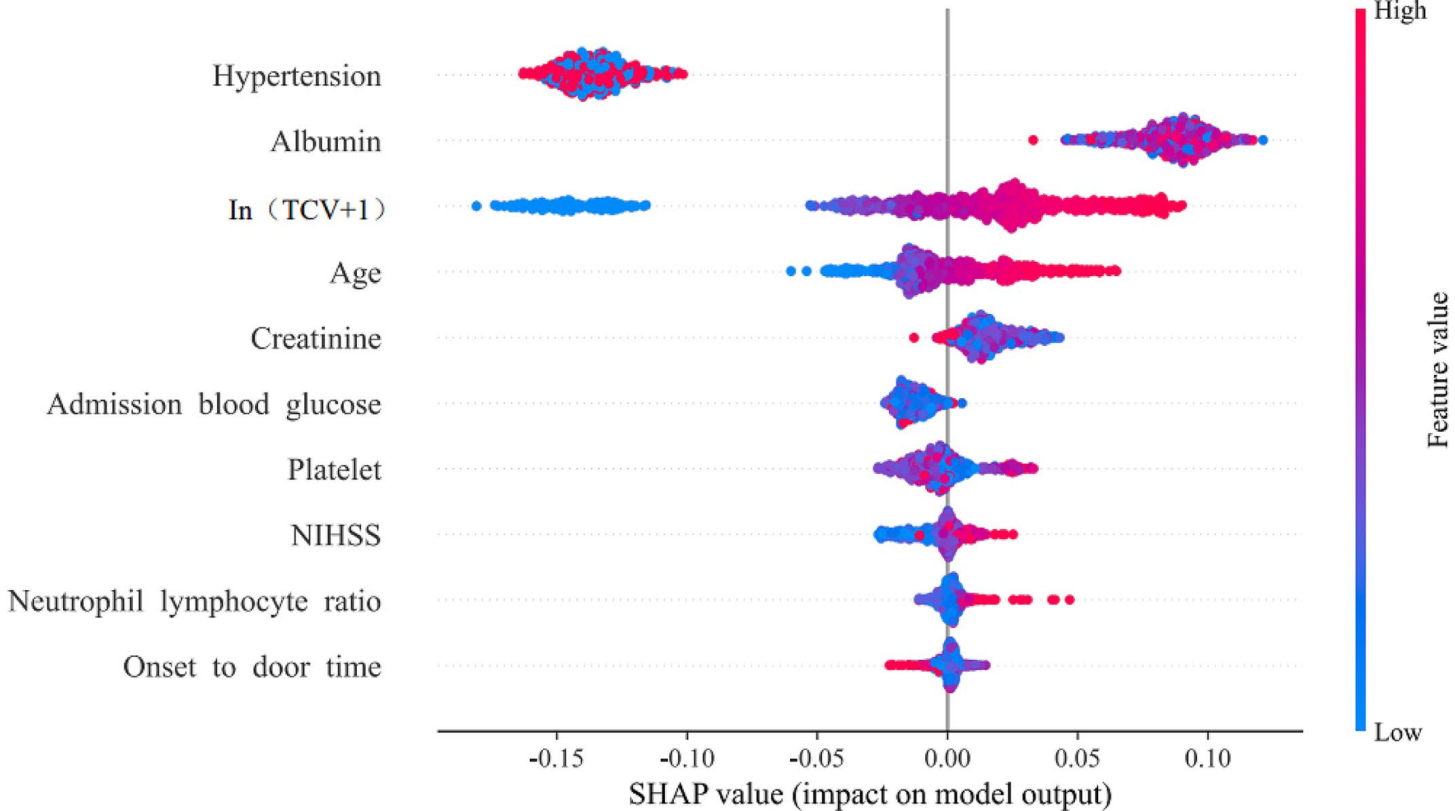
SHAP swarm plot for feature influence.

The Extra Trees model was identified as the optimal model through a comprehensive evaluation that integrated statistical comparison of AUC via DeLong’s test, alongside assessments of accuracy, sensitivity, and specificity (Supplementary Figures 1, 2). This model achieved an AUC of 0.89 in the training set, with an accuracy of 0.81, 0.82, and 0.80, respectively. In the internal validation set, it achieved an AUC of 0.85, with an accuracy of 0.80, sensitivity of 0.79, and specificity of 0.81. SHAP analysis illustrated the directional contribution of each predictor. Figure 5 displays SHAP values for the ten predictors ranked by the mean absolute contribution. Feature values (blue = low, red = high) indicate the direction of impact on prediction outcomes. The three most influential predictors were history of hypertension, serum albumin level, and TCV, followed by age, creatinine, admission blood glucose, platelet count, NIHSS, neutrophil-to-lymphocyte ratio, and onset-to-door time.

### 3.4 External validation

**Figure 6:**
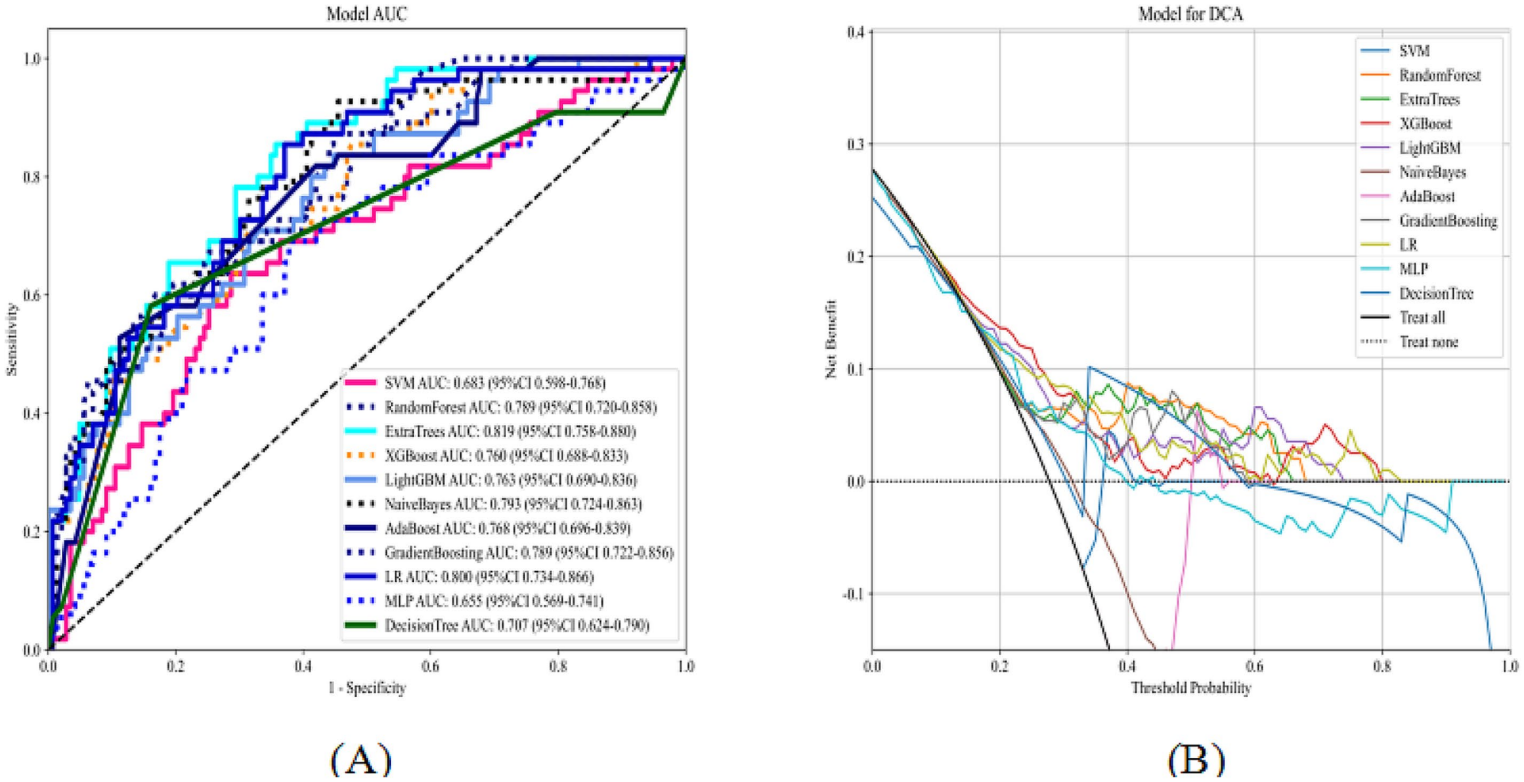
ROC curves and DCA of 11 machine learning models on the external test set (**A**: ROC curves; **B**: DCA).

Figure 6 shows the ROC and decision curves for the external test set across the 11 models. The AUC, accuracy, sensitivity, and specificity values for the different models are summarized in Supplementary Table 2. For the external test set, the optimal model based on Extra Trees achieved an AUC of 0.82 (95% confidence interval: 0.76–0.88), with accuracies of 0.70, 0.85, and 0.64. These results confirmed the model’s strong predictive and generalization capabilities. DCA demonstrated a substantial net clinical benefit of using the model to guide MT decisions across a broad range of threshold probabilities, indicating high clinical applicability.

## 4 Discussion

The machine learning models developed and validated in this study, based on preoperative clinical data and imaging features of arterial calcification, demonstrated strong predictive performance. To our knowledge, this is the first study to incorporate preoperative emergency data–including IAC–into machine learning frameworks to predict MT outcomes. By limiting feature selection to preoperative data, the models maximized clinical applicability and provided actionable guidance for emergency physicians in making personalized diagnostic and therapeutic decisions.

Among the 11 machine learning models constructed, the Extra Trees model demonstrated superior predictive performance, achieving AUC values exceeding 0.8 across the training, internal validation, and external test sets. The primary advantage of machine learning models over traditional logistic regression lies in their ability to automatically capture nonlinear relationships and feature interactions, thereby reducing collinearity effects and exhibiting stronger predictive capabilities in high-dimensional data (22). The Extra Trees algorithm employs ensemble learning with decision trees, balancing performance and efficiency in binary classification tasks through dual randomness–random feature selection and random split points–making them particularly more suited for handling high-dimensional noise and interaction effects in complex medical data (23). To enhance interpretability, the SHAP method was used to explain the model results and demonstrate feature importance rankings. The three most critical predictors were a history of hypertension, serum albumin level, and TCV, followed by age, creatinine level, admission blood glucose level, platelet count, NIHSS score, neutrophil-to-lymphocyte ratio, and onset-to-admission time. Among these, age and NIHSS score were commonly included in previous scoring systems, while emergency admission blood glucose aligned with the HIAT score metrics. Both the HIAT2 score and the machine learning model constructed by Nishi et al. (15) included only the simplified semi-quantitative imaging indicator ASPECTS, without incorporating advanced neuroimaging features. The results of this study suggest that TCV evaluation of IAC is a more suitable imaging predictor than ASPECTS. Hypertension, a traditional risk factor for cerebrovascular disease, may influence MT outcomes through multiple mechanisms (24). Prolonged, uncontrolled preoperative hypertension can exacerbate atherosclerosis, increase vascular tortuosity and thrombus burden, and thereby hinder the passage of thrombectomy devices and prolong procedural times. Intraoperative and postoperative hypertension may trigger cerebral hyperperfusion syndrome, leading to blood–brain barrier disruption, increased risk of brain edema, and a higher probability of hemorrhagic transformation. Albumin may exert protective effects on MT outcomes through mechanisms such as antioxidative stress, anti-inflammation, maintenance of osmotic pressure, protection of vascular endothelial function, and stabilization of blood–brain barrier integrity (25). In Yao et al.’s (12) MT machine-learning prediction model, which was based on postoperative laboratory indicators, albumin was the core predictor. This study further validated the predictive role of albumin level using preoperative data. The remaining model features consisted of routine clinical characteristics and laboratory results available in most hospitals, thereby ensuring clinical practicality.

IAC was previously considered a marker of irreversible vascular aging; however, recent studies have revealed that it involves multiple pathophysiological mechanisms and represents a dynamically regulated process (26) that closely associated with the onset, development, and prognosis of AIS (27, 28). Vascular lumen stenosis and hardening caused by calcification may more directly influence MT treatment outcomes in patients with AIS. Nevertheless, current research conclusions remain inconsistent. A semiquantitative study conducted by Haussen et al. (17) suggested that extensive calcification of the intracranial internal carotid artery does not affect the clinical outcomes of endovascular treatment. In contrast, Hernández-Pérez et al. (29) reported that intracranial carotid artery calcification volume was associated with MT outcomes at 90 days post-treatment, based on a cohort of 194 patients who were either unresponsive to thrombolytic therapy or had contraindications. Compagne et al. (30) conducted a *post hoc* subgroup analysis of 344 patients with anterior circulation stroke from the 2015 MR CLEAN trial, finding better efficacy in those with medial calcification patterns, though no significant correlation between IAC volume and outcomes. A 2023 study by Rodrigo-Gisbert et al. involving 393 patients with AIS qualitatively indicated that symptomatic IAC could predict endovascular treatment outcomes (18). A recent prospective study by Sierra-Gómez et al. (31), utilizing a semi-quantitative visual calcification score, indicated that carotid artery calcification was associated with larger infarct volumes and poorer outcomes following MT. Despite numerous studies, the conclusions remain heterogeneous, with most relying on qualitative or semi-quantitative assessment and lacking comprehensive quantitative evaluation of IAC indicators. In this study, we conducted a thorough quantitative assessment of IAC, identifying it as an important predictor of MT outcomes. These findings not only clarify the relationship between IAC and MT success but also offer valuable insights for constructing high-performance machine learning prediction models incorporating plaque imaging features.

The potential mechanisms underlying the predictive value of IAC involve several pathways, including a direct increase in plaque hardness as a marker of atherosclerosis, thereby contributing to increased surgical difficulty (32). Additionally, microcalcifications arising from the necrosis or apoptosis of lipid core cells may increase the risk of plaque rupture (33). Additionally, IAC may impair vascular endothelial function and reduce the vessel’s buffering capacity and compensatory capacities in response to blood flow changes (34, 35). The superiority of TCV over ICV and other calcification metrics is attributed to its more accurate reflection of overall IAC distribution. In large vessel occlusion of the anterior circulation, cerebral blood flow critically depends on collateral supply from the contralateral anterior circulation and the posterior circulation via the anterior and posterior communicating arteries. Thus, assessing calcification in all major intracranial arteries, including the contralateral anterior circulation arteries, the vertebral arteries, and the basilar arteries, provides a more comprehensive evaluation of the impact of IAC on collateral compensatory flow compared to conventional approaches that evaluate only the occluded vessel.

The machine learning models developed in this study can assist physicians in predicting treatment outcomes more accurately, this enabling more personalized treatment plans. However, our study had some limitations. First, as a retrospective study, it may be subject to inherent biases. Second, the study included a relatively small number of patients in the model development cohort. Third, although internal and external validation was performed, the model’s generalizability requires further testing in diverse populations (e.g., across different races or countries). Future large-scale, multicenter prospective studies across various cohorts, including non-East Asian populations, are needed to refine and optimize the prediction models. Despite these limitations, establishing a reliable model for predicting MT outcomes holds significant value for guiding clinical decision-making.

In conclusion, total IAC volume is a critical predictor of MT outcomes in patients with anterior circulation AIS. The machine learning models developed using preoperative emergency data demonstrated strong predictive performance, offering a robust theoretical foundation for clinicians to more accurately identify patients with AIS who are suitable candidates for MT.

## Data Availability

The original contributions presented in the study are included in the article/Supplementary material, further inquiries can be directed to the corresponding author.
