# Supplementary material for: Machine learning models integrating intracranial artery calcification to predict outcomes of mechanical thrombectomy
Source: Front Neurol. 2025 Aug 6;16:1642807. doi: 10.3389/fneur.2025.1642807 (PMC12368346; doi:10.3389/fneur.2025.1642807)
Supplement: Supplementary file 1 [file Data_Sheet_1.pdf]

Supplementary materials

Supplementary Table 1. Consistency test of Intraclass Correlation Coefficient (ICC).

Items	ICC	95%CI	F	*P*
Inter-group	0.92	0.91~0.94	25.72	＜0.001

Supplementary Table 2. Predictive performance of 11 machine learning models on the external test set.

Model	AUC	Accuracy	Sensitivity	Specificity
SVM	0.68	0.69	0.64	0.71
RandomForest	0.79	0.77	0.60	0.83
ExtraTrees	**0.82**	0.70	0.85	0.64
XGBoost	0.76	0.69	0.71	0.69
LightGBM	0.76	0.63	0.84	0.55
NaiveBayes	0.79	0.65	0.93	0.55
AdaBoost	0.77	0.79	0.53	0.89
GradientBoosting	0.79	0.75	0.62	0.80
LR	0.80	0.69	0.85	0.63
MLP	0.65	0.64	0.69	0.62
DecisionTree	0.71	0.77	0.58	0.84

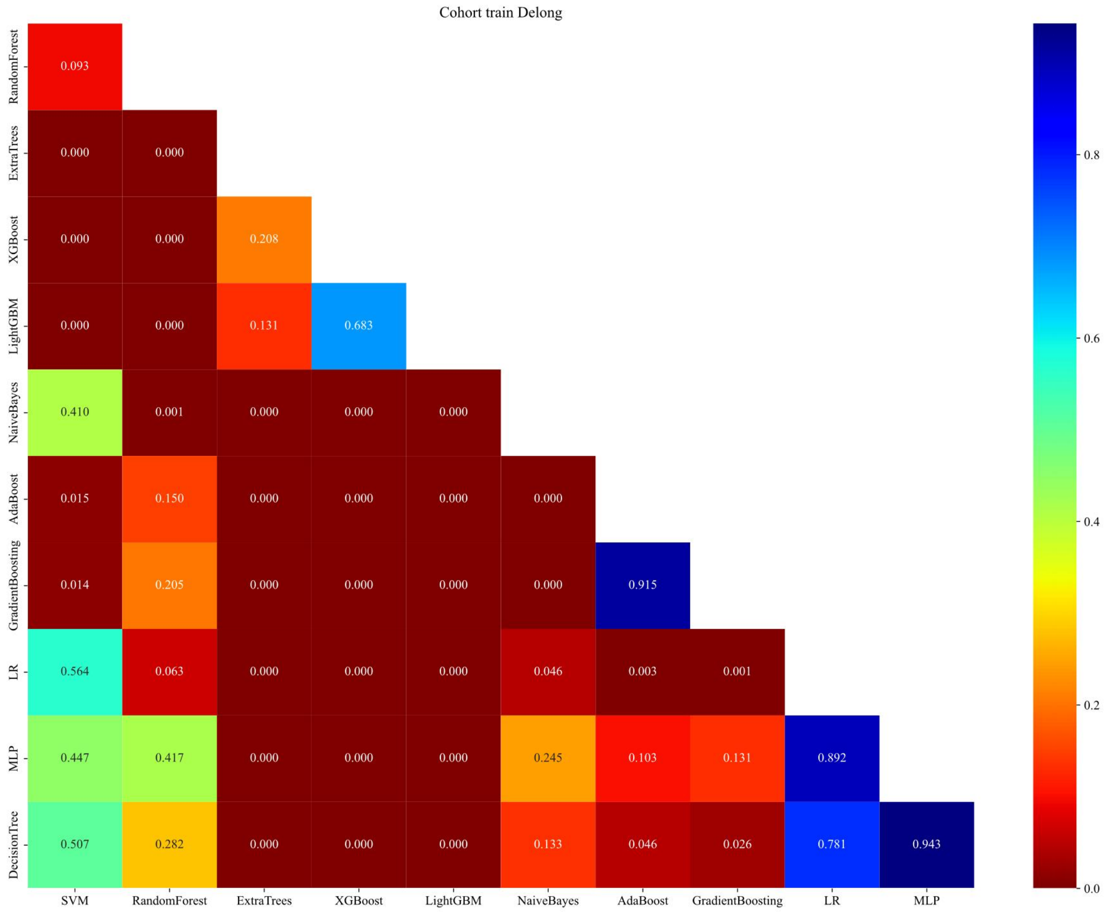

Supplementary Figure 1. DeLong's test for comparing AUCs of ROC curves of 11 machine learning models on the training set (a *P*-value less than 0.1 was considered indicative of statistical significance in DeLong's test).

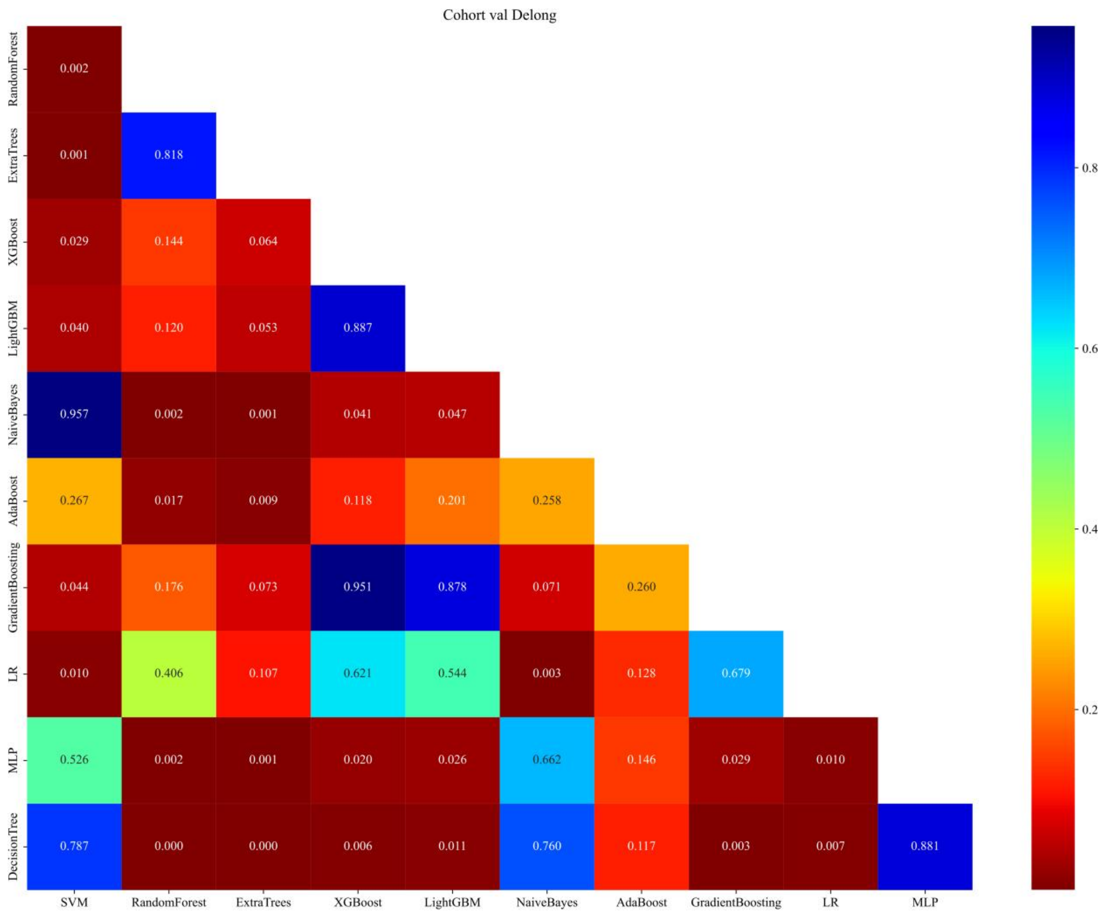

Supplementary Figure 2. DeLong's test for comparing AUCs of ROC curves of 11 machine learning models on the internal validation set (a *P*-value less than 0.1 was considered indicative of statistical significance in DeLong's test).
